# The Effects of a Chatbot-Based Interpretation Bias Modification on Early Adulthood Depression

**DOI:** 10.1192/j.eurpsy.2024.1105

**Published:** 2024-08-27

**Authors:** J. Lee, D. Lee

**Affiliations:** Kangwon National University, Chuncheon, Korea, Republic Of

## Abstract

**Introduction:**

Depression, particularly in early adulthood, presents a significant mental health challenge with far-reaching implications. Innovative approaches to address and alleviate depressive symptoms are of paramount importance in this context. One such approach involves the utilization of technology, specifically chatbot-based programs, to target specific cognitive biases associated with depression.

**Objectives:**

The central objective is to empirically examine whether this program can effectively influence depressive mood and negative cognition in individuals grappling with depressive symptoms.

**Methods:**

To ascertain the program’s efficacy, participants were divided into two groups: the CBM-I group (n=20), which underwent interpretation bias modification training, and the Mood Check group(n=20), which served as a control and engaged in a simple mood-checking exercise. A battery of psychological measures was employed, including assessments of depression, interpretation bias, suicidal ideation, resilience, and attention control.

**Results:**

Analysis results showed that the CBM-I group had a significant reduction in depression (PHQ-9, CES-D) compared to the Mood Check group in the post-measurement. Moreover, resilience (CD-RISC) and attention control (ACQ) significantly improved in the CBM-I group.

**Conclusions:**

This research serves as a stepping stone towards a deeper understanding of how chatbot-based interventions can contribute to the management of early adulthood depression, offering new perspectives and possibilities in the realm of mental health support and treatment.

**Disclosure of Interest:**

None Declared

